# The fission yeast methyl phosphate capping enzyme Bmc1 guides 2′-O-methylation of the U6 snRNA

**DOI:** 10.1093/nar/gkad563

**Published:** 2023-07-05

**Authors:** Jennifer Porat, Viktor A Slat, Stephen D Rader, Mark A Bayfield

**Affiliations:** Department of Biology, York University, Toronto, Canada; Department of Biochemistry and Molecular Biology, University of British Columbia, Vancouver, Canada; Department of Biochemistry and Molecular Biology, University of British Columbia, Vancouver, Canada; Department of Chemistry and Biochemistry, University of Northern British Columbia, Prince George, Canada; Department of Biology, York University, Toronto, Canada

## Abstract

Splicing requires the tight coordination of dynamic spliceosomal RNAs and proteins. U6 is the only spliceosomal RNA transcribed by RNA Polymerase III and undergoes an extensive maturation process. In humans and fission yeast, this includes addition of a 5′ γ-monomethyl phosphate cap by members of the Bin3/MePCE family as well as snoRNA guided 2′-O-methylation. Previously, we have shown that the Bin3/MePCE homolog Bmc1 is recruited to the *S. pombe* telomerase holoenzyme by the LARP7 family protein Pof8, where it acts in a catalytic-independent manner to protect the telomerase RNA and facilitate holoenzyme assembly. Here, we show that Bmc1 and Pof8 are required for the formation of a distinct U6 snRNP that promotes 2′-O-methylation of U6, and identify a non-canonical snoRNA that guides this methylation. We also show that the 5′ γ-monomethyl phosphate capping activity of Bmc1 is not required for its role in promoting snoRNA guided 2′-O-methylation, and that this role relies on different regions of Pof8 from those required for Pof8 function in telomerase. Our results are consistent with a novel role for Bmc1/MePCE family members in stimulating 2′-O-methylation and a more general role for Bmc1 and Pof8 in guiding noncoding RNP assembly beyond the telomerase RNP.

## Graphical Abstract

**Graphical Abstract:**
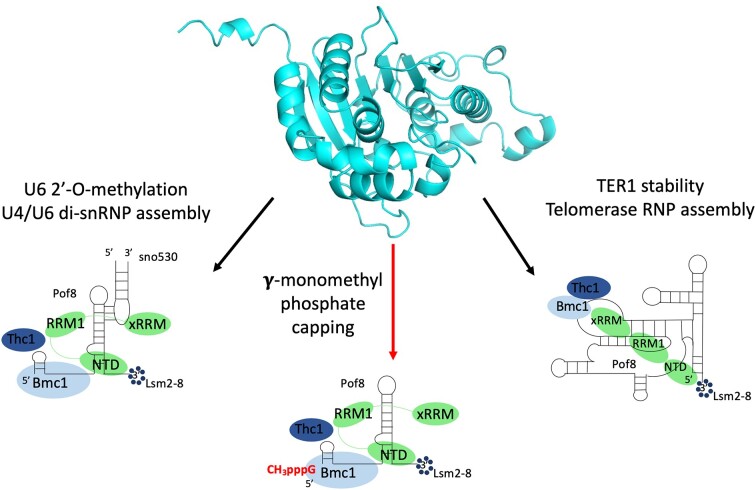

## INTRODUCTION

Pre-mRNA splicing, comprised of intron excision and subsequent exon ligation, relies on dynamic RNA-RNA and RNA-protein interactions in the spliceosome (reviewed in (1)). The spliceosome contains upwards of 100 proteins (2) and 5 uridylate-rich small nuclear RNAs (snRNAs): U1, U2, U4, U5 and U6. The U6 snRNA, which forms part of the catalytic core of the spliceosome (3), undergoes several conformational changes during pre-spliceosome assembly and splicing catalysis, which enables its interaction with other spliceosomal RNAs and the switch between a catalytically active and inactive state (4). As such, U6 biogenesis and maturation is complex and tightly regulated to ensure correct functioning in the spliceosome (reviewed in (5)).

In addition to being the most highly conserved of the snRNAs, U6 is also the only snRNA transcribed by RNA Polymerase III (RNAP III) (6). In humans and *Schizosaccharomyces pombe*, transcription of U6 by RNAP III is associated with the addition of a 5′ γ-monomethyl phosphate cap catalyzed by enzymes of the Bin3/MePCE (methyl phosphate capping enzyme) family (7–9). U6 contains a 5′ stem loop critical for 5′ capping (10), as well as an internal stem loop (ISL) that forms during splicing catalysis. The ISL is mutually exclusive with U4/U6 base pairing that occurs in pre-spliceosome snRNPs (11). U6 also contains 2′-O-methylated, pseudouridylated, and m6A-modified nucleotides, with pseudouridines largely present towards the 5′ end and 2′-O-methylations tending to cluster in the ISL (12,13). Moreover, U6 maturation in fission yeast involves the splicing of an mRNA-type intron, thought to arise from reverse splicing, as the intron is located near the catalytic nucleotides of U6 (14–18). Most information about the timing of U6 processing events has come from elegant studies in budding yeast (reviewed in (5)). However, since budding yeast U6 lacks 2′-O-methylations and a Bmc1 homolog (19,20), several questions remain as to the timing and importance of post-transcriptional modifications with respect to other U6 processing steps in organisms like fission yeast and humans.

In addition to 5′ γ-monomethyl phosphate capping enzymes, several other proteins have been linked to U6 processing. These include the La protein, which associates with nascent U6 transcripts through the 3′ uridylate tail (21), and the Lsm2-8 complex, which binds end-matured U6 and remains stably associated through spliceosome assembly (22–24). Recent work revealed that mammalian LARP7, a La-Related Protein (LARP) previously linked to MePCE in the context of the 7SK snRNP (25), is also involved in post-transcriptional processing of U6. LARP7 promotes 2′-O-methylation of U6 by the methyltransferase fibrillarin, which in turn contributes to splicing fidelity at elevated temperatures in humans and in male germ cells in mice (26,27). Conversely, ciliate and fission yeast LARP7 homologs have been well studied for their roles in telomerase biogenesis (28–33). We and others have reported that the *S. pombe* LARP7 protein Pof8 associates with the Bin3/MePCE homolog Bmc1 and a fission yeast-specific protein, the telomerase holoenzyme component 1 (Thc1) (20,34), which shares homology with the nuclear cap binding complex and poly-adenosine ribonuclease, both of which are involved in human telomerase RNA biogenesis (35,36). We have also shown that the Bmc1/Pof8 interaction is important for optimal telomerase activity, and that the link between these proteins is evolutionarily conserved across diverse fungal species (19,20,34). Thus, while much has been learned about MePCE/Bmc1 function in the 7SK snRNP and telomerase, its precise role in U6 biogenesis and function remains unknown.

In this work, we set out to examine the role of Bmc1 in U6 biogenesis and spliceosome function. We describe a new RNP containing the U6 snRNA and the telomerase components Bmc1, Pof8 and Thc1, and show that this complex is required for wild type levels of 2′-O-methylation in the U6 ISL and U6 snRNP assembly. We also show that while Bmc1’s 5′ capping catalytic activity is not required for its function in promoting 2′-O-methylation of U6, an intact Pof8-Lsm2-8 interaction is. Finally, we show that Bmc1 deletion influences the splicing of some introns under wild-type and heat-shock conditions, consistent with previous work linking human LARP7 to splicing robustness. Using our transcriptome-wide pre-mRNA splicing data set, we also show that increased intron retention upon heat shock in *S. pombe* is linked to particular intron features including strength of 5′ splice site sequences. Together, these data point towards an intricate network of post-transcriptional processing events that are critical for normal U6 biogenesis, and provide the first direct evidence for a function of the Bin3/MePCE family in promoting U6 snRNP maturation.

## MATERIALS AND METHODS

### Yeast strains and growth

Strains were grown at 32°C in yeast extract with supplements (YES) or Edinburgh Minimal Media (EMM), as indicated. Tag integration and knockouts were generated as described in (20) (primer sequences provided in Supplementary Table S1). A list of yeast strains is provided in Supplementary Table S2.

### Native protein extracts and immunoprecipitation

Native protein extractions and immunoprecipitations were carried out as described in (20). Protein A-tagged strains were immunoprecipitated with Rabbit IgG-conjugated (MP-Biomedicals, SKU 085594) Dynabeads (Invitrogen, 14301) (37) and myc- and HA-tagged proteins were immunoprecipitated with Protein A/G beads (GeneBio, 22202B-1) coated with anti-myc antibody (Cell signaling, 2276S) at a dilution of 1:250 or anti-HA antibody (Cell signaling, 3724S) at a dilution of 1:50. Total RNA was isolated from cell extracts with 0.5% SDS, 0.2 mg/ml Proteinase K (Sigma, P2308), 20 mM Tris–HCl pH 7.5, and 10 mM EDTA pH 8.0 for 15 min at 50°C, followed by phenol: chloroform: isoamyl (25:24:1) extraction and ethanol precipitation. Immunoprecipitated RNA was isolated by incubating beads in 0.1% SDS and 0.2 mg/ml Proteinase K for 30 min at 37°C, followed by phenol: chloroform: isoamyl alcohol extraction. For native northern blots, input RNA was extracted in the same manner as immunoprecipitated RNA. Relative immunoprecipitation efficiency was calculated by dividing the IP signal by the input signal. Western blots were performed using anti-myc (Cell signaling, 2276S) at 1:5000, anti-beta actin (Abcam, ab8226) at 1:1250, HRP-conjugated anti-mouse (Cell signaling, 7076) at 1:5000, anti-HA (Cell signaling, 3724S) at 1:1000, HRP-conjugated anti-rabbit (Cell signaling, 7074S) at 1:5000, or HRP-conjugated polyclonal anti-Protein A (Invitrogen, PA1-26853) at 1:5000.

### RNA preparation, northern blotting, 2′-O-methylation detection, and solution hybridization

Total RNA was extracted with hot phenol, separated on 10% TBE-urea polyacrylamide gels, and transferred to positively charged nylon membranes (Perkin Elmer, NEF988001) as per (38). For native RNA extraction to detect U4/U6 duplexes, RNA was extracted with cold phenol, as per (39). Solution hybridization was performed as per (40) and resolved on 9% TBE gels. Probe sequences for ^32^P γ-ATP-labeled DNA probes for northern blotting are provided in Supplementary Table S3. Primer extensions to detect 2′-O-methylation were performed based on protocols from (41). Briefly, 5 μg RNA was incubated for 5 min at 85°C in a 10 μl reaction containing ^32^P γ-ATP-labeled probe, 50 mM Tris–HCl pH 7.4, 60 mM NaCl, then transferred to 55°C for 20 min to allow the probe to anneal. Reverse transcription was carried out with 1.5 mM (high concentration) or 0.1 mM (limiting concentration) dNTP mix and 2.5 U AMV-RT (NEB, M0277S) and 1 h incubation at 42°C. cDNA products were separated on 8% TBE–urea sequencing gels, dried, and exposed to Phosphor screens overnight. Relative 2′-O-methylation was calculated by determining the ratio of each RT stop relative to the total signal in each lane (all RT stops and full length U6). 2′-O-methylations were also detected by RNase H (NEB, M0297S) digestion of 2 μg with 25 pmol chimeric RNA-DNA probes, as per (42). Probe sequences targeting C57 and A64 2′-O-methylations are provided in Supplementary Table S3.

### qRT-PCR and semi-quantitative RT-PCR

1 μg TURBO DNase-treated RNA was reverse transcribed with the iScript cDNA reverse transcription kit (Biorad, 1708890) or 5 U AMV-RT (NEB, M0277S) and gene-specific reverse primers. qRT-PCR was performed with the SensiFAST SYBR No-Rox kit (Bioline, BIO-98005) and 1 μM of each primer, with settings outlined in (20). For semi-quantitative RT-PCR, cDNA was amplified with Taq polymerase (NEB, MO273L) using the following cycling conditions: 5 min initial denaturation at 94°C, 26 (pud1, alp41) or 27 (rpl1603, bor1) cycles of 30 s at 94°C, 30 s at 50°C, and 1 min at 72°C, and a final 5 min extension at 72°C. cDNA was resolved on 10% TBE gels.

### Native yeast extract preparation, native snRNP gels and glycerol gradient sedimentation

Pellets from 1 L yeast cultures were resuspended to 1 g/ml in AGK400 buffer (10 mM HEPES–KOH pH 7.9, 400 mM KCl, 1.5 mM MgCl_2_, 0.5 mM DTT, 1 mM PMSF, and protease inhibitor cocktail (Sigma, P8215)), frozen in liquid nitrogen, and ground to fine powder with a mortar and pestle. Powder was thawed on ice and spun in a JA 25.50 rotor (Beckman) for 16 min at 15000 rpm and the supernatant was subsequently spun in a 70.1 Ti rotor (Beckman) for 45 min at 50 000 rpm to pellet ribosomes and heavy molecular weight complexes. Supernatants were flash frozen and stored at −80°C. For native snRNP gels, glycerol with xylene cyanol and bromophenol blue was added to 30 μg cell extract (final glycerol concentration = 10%) and fractionated on 4% 19:1 acrylamide: bis-acrylamide native gels (15 cm × 18 cm) for 220 min at 240 V and 4°C, then transferred to nylon membranes for northern blotting. For glycerol gradients, cell extracts from 1.0 g frozen cell powder were layered on an 11 ml 10–30% glycerol gradient (50 mM Tris–HCl pH 7.4, 25 mM NaCl, 5 mM MgCl_2_) and spun in an SW41Ti rotor (Beckman) for 20 hours at 30 900 rpm. Fractions were collected starting from the top of the gradient and RNA and proteins were extracted with phenol: chloroform: isoamyl alcohol (25:24:1) and TCA precipitation, respectively.

### UV melt curves

UV melt curves were recorded on a Cary BIO 100 spectrometer with a 6 × 6 temperature-controlled cell holder. 2 μl 10 mM U4 and modified or unmodified U6 RNA oligos in 96 μl buffer (10 mM KH_2_PO_4_ pH 7.0 and 200 mM KCl) was heated and cooled from 50°C to 65°C at a rate of 2°C per minute without collecting data, then re-heated and cooled while monitoring absorbance at 260 nm at 1°C intervals. Absorbance at 260 nm at each temperature point was normalized to absorbance at 50°C and absorbance curves were fitted with an equation for one site specific binding with a Hill slope to determine *T*_m_ values. RNA sequences are provided in Supplementary Table S3.

### RNA seq and intron retention analysis

DNase-treated RNA was rRNA-depleted (Qiagen, 334215) and stranded libraries were prepared by Genome Québec. cDNA libraries were sequenced on a NovaSeq6000 with 150 bp paired-end reads. Reads were aligned to the fission yeast genome (ASM294v2) with Bowtie2 (43). Intron retention was quantified using IRFinder (version 2.0.1), as per (44,45). Any introns flagged as having a low sequencing depth or fewer than 4 reads to support splicing were not considered for statistical analysis. Differential intron retention was calculated using DESeq2 (46 ). Sequence extraction for *S. pombe* introns was carried out using BEDTools v2.3.0 (47) and sequences are provided in dataset 1. 5′ splice sites (3 bases in the exon and 6 bases in the intron) and 3′ splice sites (20 bases in the intron and 3 bases in the exon) were scored with MaxEntScan using a maximum entropy model (48). Intron free energy of the thermodynamic ensemble (kcal/mol) was calculated using RNAfold v2.5.1 (49).

## RESULTS

### Bmc1 forms a U6-containing complex with the telomerase proteins Pof8 and Thc1

**Figure 1.**
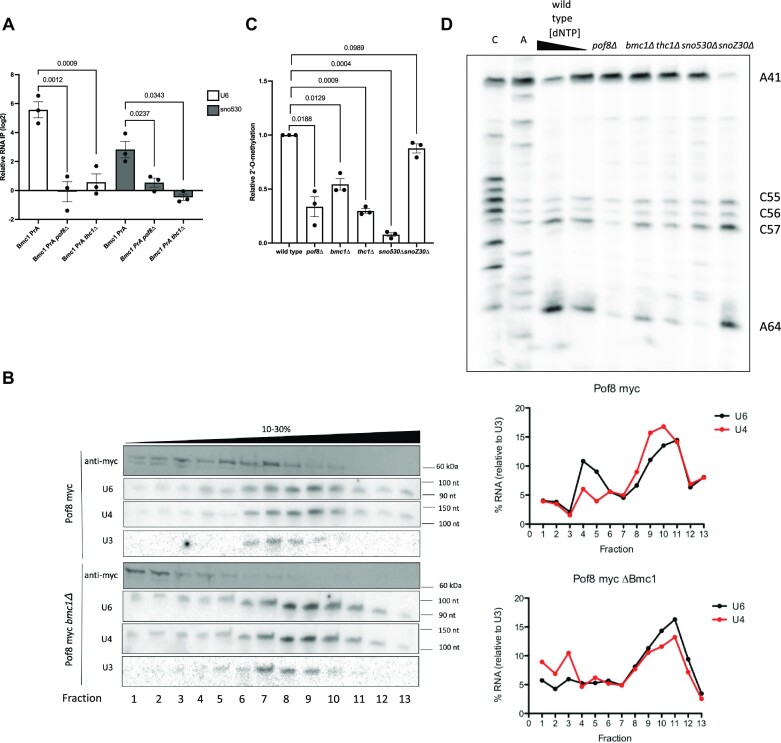
Bmc1, Pof8, and Thc1 guide 2′-O-methylation of U6. (**A**) qRT-PCR of U6 and sno530 in Bmc1 PrA immunoprecipitates, normalized to immunoprecipitation from an untagged strain (mean ± standard error, two-tailed unpaired *t* test) (*n* = 3 biological replicates). (**B**) Glycerol gradient sedimentation of myc-tagged Pof8, U4 and U6, and U3 from wild type (Pof8 myc) and *bmc1Δ* strains. U4 and U6 signals were normalized to U3 for calculating relative migration in the gradient. (**C**) Quantification of relative 2′-O-methylation-induced reverse transcriptase stops at A64, compared to a wild type strain (mean ± standard error, two-tailed paired *t* test) (*n* = 3 biological replicates). (**D**) 2′-O-methylation primer extension of U6 at high (1.5 mM) and limiting (0.1 mM) dNTP concentrations. 2′-O-methylated sites are indicated.

Our previous work characterizing Bmc1 as a component of the telomerase holoenzyme also revealed interactions between Bmc1 and various other noncoding RNAs, including the U6 snRNA (Supplementary Figure S1A) (20). We therefore tested whether Bmc1 has a role in the biogenesis, stability, or function of these transcripts, and if this function is linked to the Bmc1-interacting telomerase components Pof8 and Thc1. Having already demonstrated that Pof8 is required to recruit Bmc1 to the telomerase RNA TER1 (20), we determined the protein binding requirements for U6. In contrast to what has been reported for TER1, for which (reduced) binding to Pof8 persists in the absence of Bmc1 (20,34), we found that all three proteins are necessary for an interaction with U6 (Figure 1A, Supplementary Figure S1B). Our results indicating an interaction between Pof8 and U6 are also consistent with previous work identifying mammalian LARP7 as a U6-interacting protein (26,27), suggesting that LARP7 family members have conserved functions related to U6, in addition to LARP7 function in telomerase in *S. pombe* and ciliates (29–31).

As an additional means to confirm U6 snRNP formation, we fractionated native cell extracts on glycerol gradients and compared protein and RNA sedimentation in wild type and knockout yeast strains (Figure 1B, Supplementary Figure S1C). A substantial fraction of Bmc1 and Pof8 co-migrated with U6, in particular toward the lighter sedimenting U6 containing fractions (lanes 2–7). Importantly, co-migration of Pof8 with U6 was lost upon deletion of Bmc1 (Figure 1B), as well as co-migration of Bmc1 with U6 upon deletion of Pof8 (Supplementary Figure S1C). We propose that Bmc1, Pof8, and Thc1 associate with U6 simultaneously, with all three proteins required to be present to initiate formation of the Bmc1-containing U6 snRNP. We also fractionated cell extracts on higher percentage glycerol gradients to compare sedimentation of the U6- and Bmc1-containing snRNP to the telomerase holoenzyme (Supplementary Figure S1D). The telomerase RNA TER1 migrated in heavier sedimenting fractions (fractions 6–9) compared to the U6 peak that was sensitive to Pof8 deletion (fractions 2–4), suggesting that the U6 and telomerase peaks are not overlapping, and thus distinct complexes.

Together, these data point towards the existence of a new U6-containing complex that also shares components with the telomerase holoenzyme, providing a surprising link between two seemingly disparate fission yeast noncoding RNA pathways.

### Bmc1, Pof8, and Thc1 promote 2′-O-methylation of U6

To gain further insight into the role of the Bmc1-containing U6 snRNP, we examined our Bmc1 RIP-Seq dataset (20), which revealed an interaction between Bmc1 and snoZ30, which guides 2′-O-methylation of U6 at position 41 (41) (Supplementary Figures S1A, S2A, B). Further supporting the idea that U6 complex formation is contingent on the presence of all three proteins, we observed a loss of snoZ30 binding to Bmc1 upon knockout of any member of the complex (Supplementary Figure S2A). The observed interaction between Bmc1 and snoZ30, coupled with the recently described function of mammalian LARP7 in facilitating snoRNA-guided 2′-O-methylation of U6 by the methyltransferase fibrillarin (26,27) provided initial clues as to the function of this new Bmc1- and U6-containing snRNP. To determine if Bmc1, Pof8, and Thc1 influence 2′-O-methylation, we mapped U6 2′-O-methylation sites by performing primer extensions at low dNTP concentrations (41). Although snoZ30 is the sole annotated U6-modifying snoRNA in fission yeast (41), several other 2′-O-methylated sites have been identified in U6, including A64 (13). Deletion of Bmc1, Pof8, and Thc1 resulted in no observable changes in 2′-O-methylation at the snoZ30-modified A41, but we did detect a reproducible decrease in modification at several other sites, most notably A64 (Figure 1C, D, Supplementary Figure S3).

Initial attempts at identification of the U6 A64-methylating snoRNA using box C/D snoRNA consensus sequences and base pairing rules (50) yielded no other obvious snoRNA candidates, so we instead turned to our Bmc1 RIP-Seq dataset in the hope we might identify novel snoRNAs (Supplementary Figure S1A). The uncharacterized fission yeast noncoding RNA, SPNCRNA.530 (henceforth referred to as sno530), contains a D box, a putative C box one nucleotide different from the C box consensus motif, and a region with 12 nucleotides of complementarity with U6, including a single non-Watson Crick base pair (Supplementary Figure S2C). It is also noteworthy that the predicted secondary structure of sno530 does not position the C and D boxes flanking a hairpin, as is common for canonical box C/D snoRNAs (Supplementary Figure S2C). We validated the interaction between Bmc1 and sno530 by RNP immunoprecipitation/qPCR and showed that much like snoZ30 and U6, this interaction is dependent on the presence of the assembled Bmc1-Pof8-Thc1 complex (Figure 1A). Deletion of snoZ30 and sno530 resulted in a loss of 2′-O-methylation at A41 and A64, respectively, suggesting that sno530 is indeed the A64 U6-modifying snoRNA (Figure 1C, D, Supplementary Figure S3). We obtained similar results using a complementary method that exploits the tendency for 2′-O-methylations to block RNase H cleavage following the annealing of a chimeric DNA-2′-O-methylated RNA oligo targeting the suspected 2′-O-methylated site (42,51) (Supplementary Figure S4A). This also served to provide evidence for 2′-O-methylation at C57, suggesting that it, too, is another site in U6 whose modification is similarly guided by Bmc1 and Pof8 (Supplementary Figure S4B). While our knockout studies unambiguously identify sno530 as the A64 U6-modifying snoRNA, the unusual sequence and architecture of sno530 relative to snoZ30 is more reminiscent of the divergent box C'/D' motifs that stimulate rRNA 2′-O-methylation by providing additional regions of complementarity surrounding the methylated site (52,53). We were unable to identify any candidate snoRNA(s) responsible for methylating C55, C56, and C57, by searching the fission yeast genome or through our Bmc1 RIP-Seq dataset, although this may be because the snoRNA does not conform to canonical snoRNA motifs or RNA-snoRNA base pairing rules.

### Bmc1, Pof8 and Thc1 are involved in U6 snRNP assembly

We then tested how disruption of the Bmc1-containing U6 snRNP might impact spliceosome assembly by fractionating cell extracts in a glycerol gradient and assaying protein and snRNA distribution. In wild-type cells, we noted a lowly abundant, lighter sedimenting complement of U6 that does not appear to co-sediment with U4 (compare U6 and U4 in lanes 3–6 in Figure 1B and Supplementary Figure S1C), suggesting a U6-containing complex outside of the more abundant U4/U6 di-snRNP. Consistent with this, we note that the migration of Pof8 and Bmc1 does not fully overlap with U4/U6 in the gradient, but is rather shifted towards lighter fractions, arguing against the inclusion of the Bmc1-Pof8-Thc1 complex in the U4/U6 di-snRNP (Figure 1B, Supplementary Figure S1C and below). As the lighter sedimenting U6 species is not evident in Bmc1 and Pof8 KO strains (compare U6 and U4 in lanes 3–6 in Figure 1B and Supplementary Figure S1C relative to Bmc1 and Pof8 KO strains), we hypothesized that Bmc1, Pof8 and Thc1 interact with U6 before the U4/U6 di-snRNP.

**Figure 2.**
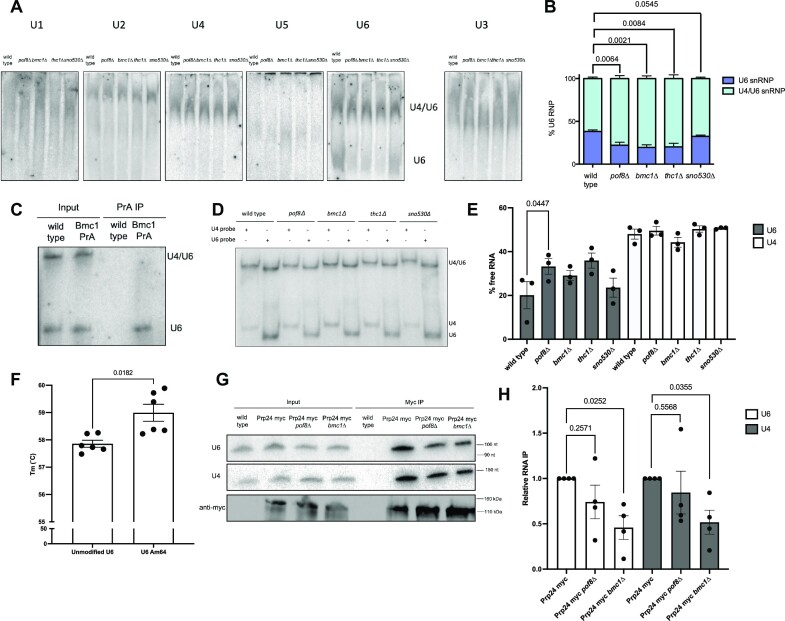
Bmc1, Pof8, and Thc1 promote U6 snRNP assembly. (**A**) Native northern blot analysis of spliceosomal and non-spliceosomal (U3) snRNPs from native yeast cell extracts. (**B**) Quantification of U6-containing snRNPs from wild type and knockout yeast cell extracts (mean ± standard error, two-tailed paired *t* test) (*n* = 4 biological replicates). (**C**) Native northern blot analysis of total and Bmc1-immunoprecipitated U6. (**D**) Solution hybridization of U4/U6 pairing in wild type and knockout yeast strains using radiolabeled probes targeting the 5′ end of U4 and 3′ end of U6. (**E**) Quantification of U4/U6 pairing from solution hybridization assay, expressed as the fraction of non-duplexed U4 and U6 (‘free RNA’) (mean ± standard error, two-tailed paired *t* test) (*n* = 3 biological replicates). (**F**) *T*_m_ values from UV melt curve analysis of U4/U6 pairing with unmodified and A64-2′-O-methylated U6 oligos (mean ± standard error, two-tailed paired *t* test) (*n* = 6 technical replicates). (**G**) Northern and western blot analysis of U4, U6 and myc-tagged Prp24 from total cell extracts and myc-immunoprecipitates. (**H**) Quantification of Prp24-immunoprecipitated U4 and U6, relative to Prp24 myc (mean ± standard error, two-tailed paired *t* test) (*n* = 4 biological replicates).

To obtain clearer resolution of distinct U6-containing complexes, we ran cell extracts on native gels and analyzed spliceosomal RNAs by northern blotting. We observed a single, prominent band for all spliceosomal RNAs except U6, which migrated as 2 distinct complexes (Figure 2A). We could assign the higher molecular weight complex, which comigrates with U4 but not U2 or U5, as the U4/U6 di-snRNP. Upon deletion of any of Bmc1, Pof8 or Thc1, we observed a significant and reproducible decrease in the intensity of the lower molecular weight U6-containing snRNP (Figure 2A, B), consistent with this band representing the Bmc1-containing U6 snRNP. The persistence of this complex upon loss of sno530 suggests that complex formation is not reliant on the ability to modify U6 at A64. Although U6 and sno530 are associated with Bmc1 (Figure 1A), sno530 is therefore not required for the stability of the U6 snRNP observed in native gels.

To understand when Bmc1 interacts with U6 with respect to spliceosome formation, we immunoprecipitated Bmc1 associated RNPs under native conditions and ran total and Bmc1-associated RNA on native gels. Bmc1 immunoprecipitated only the lower molecular weight U6 snRNP and not the species that co-migrates with the U4/U6 di-snRNP (Figure 2C), in line with the Bmc1/Pof8/Thc1-associated mono U6 snRNP band (Figure 2A, B). Based on these results, we hypothesize that the Bmc1-containing U6 complex, which promotes 5′ capping and 2′-O-methylation, is distinct from the U4/U6 di-snRNP.

Native fission yeast cell extracts do not form detectable amounts of the U4/U6.U5 tri-snRNP (54,55), so we focused our further efforts on examining U4/U6 base pairing by performing a solution hybridization assay on cold phenol-extracted total RNA to maintain U4/U6 base pairing (Figure 2D). This differs from native spliceosomal snRNP gels (Figure 2A) in that it only assesses RNA-RNA interactions, without changes in mobility due to protein binding. We detected minor defects in U4/U6 assembly upon Bmc1, Pof8, or Thc1 deletion, as measured by the increase in ‘free’ U4 relative to U4 complexed in the di-snRNP, although the increase in the fraction of free U4 only reached statistical significance upon Pof8 deletion (Figure 2D, E). Consistent with the increase in free U4 in the knockout strains, glycerol gradients revealed an increase in lighter sedimenting U4 in the knockout strains (Figure 1B, Supplementary Figure S1B, compare lanes 1–3 in wild type versus knockouts). Although U6 is in excess over U4, the increase of free U4 in the knockouts suggests that the absence of Bmc1, Pof8 and Thc1 may result in a non-functional, alternate pathway for U4 that does not involve U4/U6 di-snRNP formation. The lack of U4/U6 pairing defects upon the loss of sno530 further suggests that it is largely the Bmc1-Pof8-Thc1 protein complex dictating U4/U6 pairing, not the single A64 2′-O-methylation. Still, UV melt analysis of the U6-interacting region of U4 and the U6 internal stem loop (ISL), with or without 2′-O-methylation of A64, revealed a slight increase in U4-U6 duplex stability with 2′-O-methylation, consistent with previous findings reporting on the stabilizing properties of 2′-O-methylation on RNA duplex formation (56–58) (Figure 2F).

Subsequent steps in spliceosome formation involve unwinding of the U6 ISL and base pairing between U4 and U6, both of which are promoted by the U4/U6 di-snRNP assembly factor Prp24 (59,60). We generated an endogenously tagged Prp24 strain and assessed the interaction between Prp24 and U4 and U6 in wild-type and Bmc1 or Pof8 deleted cells. Pof8 and Bmc1 deletion resulted in a decreased interaction between Prp24 and U4 and U6, suggesting that Bmc1 and Pof8 promote the association of U4 and U6 with Prp24, which in turn may promote the formation of the U4/U6 di-snRNP (Figure 2G, H). In sum, our results are consistent with the existence of a Bmc1/Pof8/Thc1-containing U6 snRNP, with Bmc1/Pof8/Thc1 dissociating from U6 during establishment of the U4/U6 di-snRNP.

### Bmc1 5′ capping catalytic activity is not required for promoting 2′-O-methylation of U6

**Figure 3.**
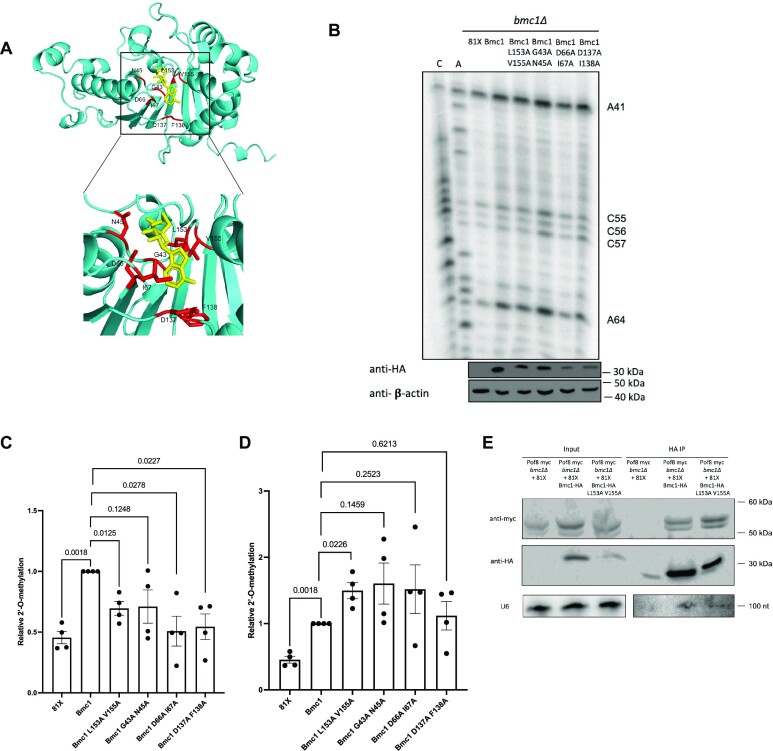
Bmc1 catalytic activity is not a requirement for 2′-O-methylation of U6. (**A**) AlphaFold (84) structure prediction of Bmc1 aligned to the SAH-bound (yellow) catalytic domain of MePCE (PDB 6DCB) (83) with mutations indicated in red. Inset: side chain interactions with SAH. (**B**) U6 2′-O-methylation primer extension in *bmc1Δ* cells transformed with the indicated plasmid. 2′-O-methylated sites are indicated. Western blots for Bmc1-HA expression and b-actin are indicated below. (**C**) Quantification of relative 2′-O-methylation-induced reverse transcriptase stops at A64, compared to wild type Bmc1-HA (mean ± standard error, two-tailed paired *t* test) (*n* = 4 biological replicates). (**D**) Quantification of relative 2′-O-methylation-induced reverse transcriptase stops at A64, compared to wild type Bmc1-HA, normalized to average Bmc1-HA expression relative to b-actin (mean ± standard error, two-tailed paired *t* test) (*n* = 4 biological replicates). (**E**) Western blot and northern blot analysis of co-immunoprecipitation of HA-tagged Bmc1, myc-tagged Pof8 and U6.

With previous studies indicating that Bmc1 5′ γ-phosphate methyltransferase catalytic activity is dispensable for telomerase activity (34), we assayed a combination of previously described and newly constructed putative Bmc1 catalytic mutants for the ability to promote U6 2′-O-methylation. We mutated residues that are both highly conserved between Bmc1 and human MePCE, and well-positioned in structure predictions to interact with the methyltransferase byproduct SAH (S-Adenosyl-L-Homocysteine) (Figure 3A). HA-tagged Bmc1 mutants were transformed into a Bmc1 knockout yeast strain and profiled for U6 2′-O-methylation as above (Figure 3B, C). While the Bmc1 mutants were more lowly expressed than wild type Bmc1, some mutants still promoted 2′-O-methylation to a greater extent than Bmc1 knockout, empty vector transformed cells (Figure 3C). Further, normalization of relative 2′-O-methylation levels to Bmc1 expression confirmed a statistically significant increase in 2′-O-methylation for all Bmc1 mutants compared to the empty vector (Figure 3D). This suggests that, as in telomerase, Bmc1 5′ γ-phosphate methyltransferase catalytic activity may not be critical for its function in promoting snoRNA-directed U6 2′-O-methylation.

For further characterization of Bmc1 catalytic mutants, we chose the Bmc1 L153A V155A mutant, which showed the highest expression across biological replicates. As measured by co-immunoprecipitation, L153A V155A still interacted with Pof8, suggesting that catalytic activity is also not required for complex formation (Figure 3E). Further, L153A V155A interacted with U6, indicating that 5′ γ-phosphate methyltransferase catalytic activity is likely not required for U6 binding (Figure 3E).

### The xRRM and Pof8-Lsm2-8 interaction are important determinants for U6 2′-O-methylation

**Figure 4.**
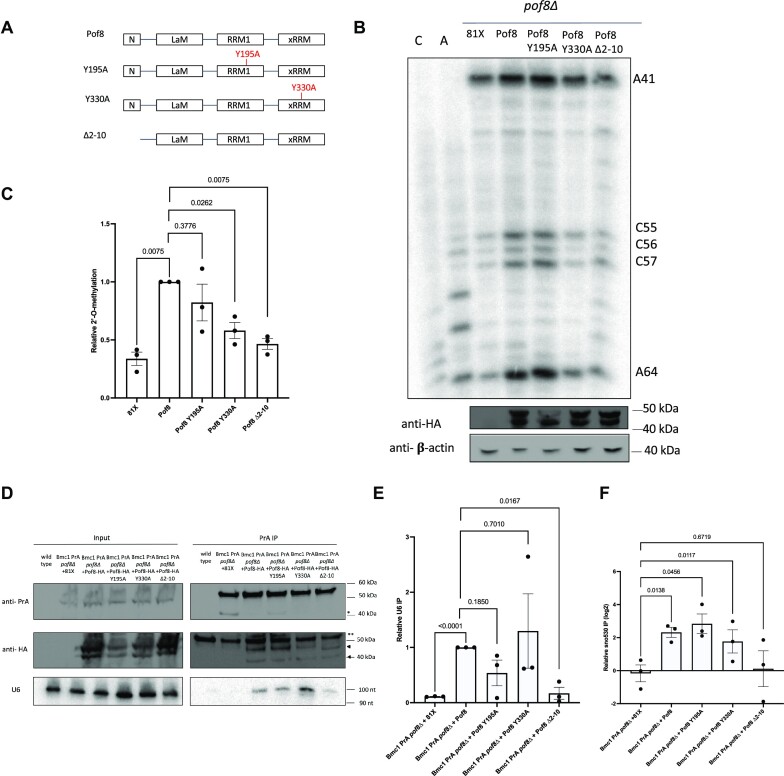
The xRRM and Lsm2-8-binding surface are important for Pof8-mediated 2′-O-methylation of U6. (**A**) Schematic of Pof8 domains and mutants used in this study. N = N-terminal domain, LaM = La motif, RRM1 = RNA Recognition Motif 1, xRRM = extended RNA Recognition Motif. (**B**) U6 2′-O-methylation primer extension in *pof8Δ* cells transformed with the indicated plasmid. 2′-O-methylated sites are indicated. Western blots for Pof8-HA expression and b-actin are indicated below. (**C**) Quantification of relative 2′-O-methylation-induced reverse transcriptase stops at A64, compared to wild type Pof8-HA (mean ± standard error, two-tailed paired *t* test) (*n* = 3 biological replicates). (**D**) Northern and western blot analysis of U6, PrA-tagged Bmc1 and HA-tagged Pof8 from total cell extracts and PrA-immunoprecipitates. *Bmc1 PrA cleavage products, **an additional band cross-reacting with the antibody, arrows indicate Pof8 HA. (**E**) Quantification of Bmc1-immunoprecipitated U6, relative to the Pof8-HA-expressing strain (mean ± standard error, two-tailed paired *t* test) (*n* = 3 biological replicates). (**F**) qRT-PCR of sno530 in Bmc1 PrA *pof8Δ* immunoprecipitates, normalized to immunoprecipitation from a strain transformed with an empty vector (mean ± standard error, two-tailed unpaired *t* test) (*n* = 3 biological replicates).

As an established member of the LARP7 family of proteins, the protein-interacting and RNA binding domains of Pof8 have been well-characterized in the context of the telomerase RNP (29–33). Pof8 contains a divergent La motif that lacks the conserved uridylate-binding residues typically seen in LARP7 proteins (31), so its interaction with the telomerase RNA TER1 is mediated by the RRM1, xRRM and the N-terminal region that makes direct protein-protein contacts to Lsm2-8, which in turn binds the uridylate-rich 3′ end of TER1 (29–32). As mutations to these regions have been shown to impair Pof8 binding to TER1 and telomere length homeostasis, we looked at the impact of these same mutations on U6 2′-O-methylation (Figure 4A). In contrast to what has been observed for TER1, where both RRMs are important for binding, only mutations to the xRRM and the Lsm2-8 binding region caused a significant reduction in 2′-O-methylation at A64 (Figure 4B, C).

To further understand the molecular basis for the drop in 2′-O-methylation, we immunoprecipitated Bmc1 in a Pof8 knockout strain re-expressing the Pof8 mutants (Figure 4D, E). Bmc1 co-immunoprecipitated all Pof8 mutants, suggesting that the 2′-O-methylation defect is not due to complete disruption of the Bmc1-Pof8 interaction (Figure 4D). The Bmc1-U6 interaction, which is dependent on the presence of Pof8 (Figure 1A), was almost completely lost in the Lsm2-8-binding mutant (Δ2–10), suggesting that its 2′-O-methylation defect may be due to a loss in U6 association with the Bmc1-Pof8-Thc1 complex (Figure 4D, E). Surprisingly, we detected no loss in U6 binding with the xRRM mutant, indicating that while U6 still interacts with the Bmc1-Pof8-Thc1 snRNP in the context of the xRRM mutant, the xRRM may have another function in facilitating U6 2′-O-methylation (Figure 4D, E). To distinguish between the possibility that the xRRM mutant is defective in snoRNA binding or another activity, we repeated Bmc1 immunoprecipitations in Pof8 knockout strains re-expressing the Pof8 mutants and analyzed sno530 by qRT-PCR (Figure 4F). Unlike human LARP7, which uses its xRRM to bind U6-modifying snoRNA (27), we only detected defects in sno530 binding with the Lsm2-8-binding mutant, not the xRRM mutant, suggesting that Pof8 may interact with snoRNAs through Lsm2-8. As the xRRM in the ciliate LARP7 protein p65 has been suggested to possess RNA chaperone activity to remodel the ciliate telomerase RNA (61,62), it is tempting to speculate that the decrease in U6 2′-O-methylation in the context of the xRRM mutant may result from defects in xRRM-mediated RNA chaperone activity, which could play a role in correctly positioning U6 and the snoRNA for 2′-O-methylation.

### Bmc1 deletion has a minor effect on pre-mRNA splicing

**Figure 5.**
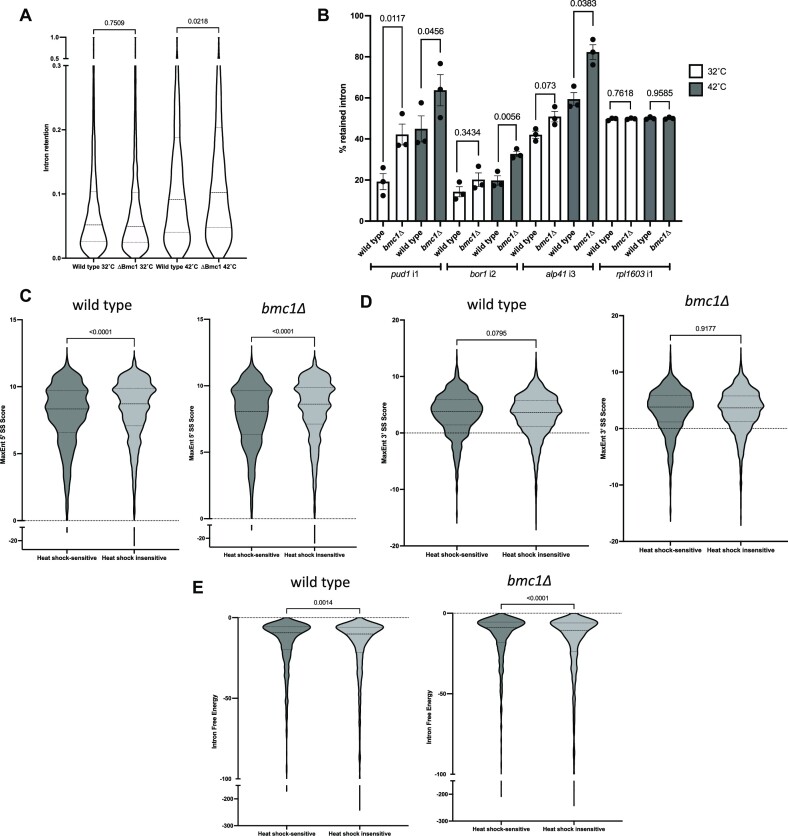
Bmc1 contributes to splicing robustness at elevated temperature. (**A**) Average intron retention from three biological replicates for wild type and *bmc1Δ* strains grown at 32°C or heat shocked at 42°C (two-tailed unpaired *t*-test with Welch's correlation). (**B**) Semi-quantitative RT-PCR in wild type and *bmc1Δ* strains grown at 32°C or heat shocked at 42°C for intron 1 of *pud1*, intron 2 of *bor1*, intron 3 of *alp41*, and intron 1 of *rpl1603* (mean ± standard error, two-tailed paired *t* test) (*n* = 3 biological replicates). Representative gels are provided in Figure S7. (C–E) Comparison of 5′ splice site scores (**C**), 3′ splice site scores (**D**) and intron minimum free energy (kcal/mol) (**E**) for heat shock-sensitive and insensitive introns in wild type and *bmc1Δ* cells. Heat shock-sensitive introns were classified as introns that exhibited a greater than 2-fold increase in intron retention following heat shock and a false discovery rate <0.05. Remaining introns are classified as heat shock insensitive. Only introns with greater than 4 reads supporting splicing in all biological replicates were included (two-tailed unpaired *t*-test with Welch's correlation).

Having observed Bmc1-dependent defects in U6 2′-O-methylation and U6 snRNP assembly, we tested the effects of Bmc1 deletion on pre-mRNA splicing. To that end, we performed short-read, paired-end sequencing on RNA extracted from wild type and Bmc1 knockout yeast strains and quantified intron retention as a proxy for splicing (44,45). We also measured intron retention in wild type and knockout cells heat shocked for 15 min at 42°C, which has been shown to impact splicing in fission yeast (63). We observed significant increases in intron retention following heat shock, similar to what has been reported in mammalian cells (64) (Supplementary Figure S5A, B). Although we observed slight increases in intron retention in Bmc1 knockout cells compared to wild type cells, very few of these splicing events at 32°C passed our significance cut-off, and no splicing events at 42°C were statistically significant (Supplementary Figure S5C, D), although this may be due to greater sample to sample variability across our triplicate replicates for this data set (Supplementary Figure S6). Still, as mean intron retention values indeed showed an increase upon Bmc1 deletion (Figure 5A), we chose several representative intron retention events to validate with semi-quantitative RT-PCR (one of which, intron 1 of *pud1*, displayed a statistically significant increase upon Bmc1 deletion at 32°C in our RNA Seq dataset). We observed an increase in intron retention following heat shock, and confirmed their further impaired splicing in the context of the Bmc1 deletion (Figure 5B, Supplementary Figure S7). Conversely, a ribosomal protein gene, which have been reported to be efficiently spliced relative to non-ribosomal protein genes in budding yeast (65,66), and did not show heat shock or Bmc1 associated changes in our RNA-Seq data set, was confirmed to have no changes in intron retention in response to heat shock or Bmc1 deletion (Figure 5B, Supplementary Figure S7). We note that these validated Bmc-1 affected introns have higher than average intron retention rates in normal cells and as such, may not be representative of the average splicing event. Together, these data indicate that Bmc1 does not have a major effect on pre-mRNA splicing, but that Bmc1 likely contributes to splicing robustness, similar to what has been described for mammalian LARP7 deletion in human cells (26,27).

We also examined other factors that could contribute to heat shock-sensitive splicing defects. We compared intronic features between heat shock-sensitive introns, classified as introns exhibiting a >2-fold increase in intron retention upon heat shock and a false discovery rate less than 0.05, and remaining introns (heat shock insensitive) (Figure 5C–E, Supplementary Figure S5A, B). We noted that heat shock-sensitive introns are enriched for weaker 5′ splice site scores in the context of both the wild type and *bmc1Δ* strains, with no significant differences in 3′ splice site scores (Figure 5C,D), in line with previous data indicating that intron retention in fission yeast is linked to weak 5′ splice sites (67),. Additionally, heat shock-sensitive introns displayed lower minimum free energy, indicative of a link between intron structure and splicing changes in response to heat shock (Figure 5E).

## DISCUSSION

### Conserved functions for LARP7 family proteins in splicing and U6 2′-O-methylation

This work represents the first report of an MePCE homolog with a role in splicing and U6 snRNP assembly, beyond 5′ methyl phosphate cap addition of U6. In our efforts to investigate functions for Bmc1 beyond telomerase, we revealed an unanticipated overlap between components of the yeast telomerase holoenzyme and a U6-containing snRNP. While it is surprising that Bmc1, Pof8, Thc1, and Lsm2-8 interact with 2 very distinct non-coding RNAs produced by different polymerases, both RNAs possess uridylate-rich sequences recognized by Lsm2-8 and highly structured regions, including stem loops in U6 and pseudoknots in telomerase, that act as scaffolds to recruit other RNP components. These common features may provide an explanation as to why these divergent RNAs share a common set of protein binding partners. Such RNP plasticity is not unique to fission yeast telomerase and U6, but may represent a shared feature of LARP7 and MePCE family proteins. Mammalian LARP7 and MePCE are particularly well-studied for their roles in capping and stabilizing the 7SK snRNP (8,25,68,69), transcriptional control through DDX21 (51), directing U6 modification (26,27), and snRNP assembly through the SMN complex (70). Thus, continuing to study the RNA interactome of MePCE and LARP7 homologs across species will likely yield additional insight into how these proteins associate with and influence various classes of non-coding RNAs. It is also possible that Bmc1, Pof8, and Thc1 interactions with U6 are mediated entirely by direct interactions with Lsm2-8, which in turn directly contacts U6, much like Prp24 interacts with U6 by directly binding Lsm2–8 (71). Future structural and biochemical studies will lend insight into the protein-protein and protein-RNA interactions that cooperate to form the U6 snRNP.

**Figure 6.**
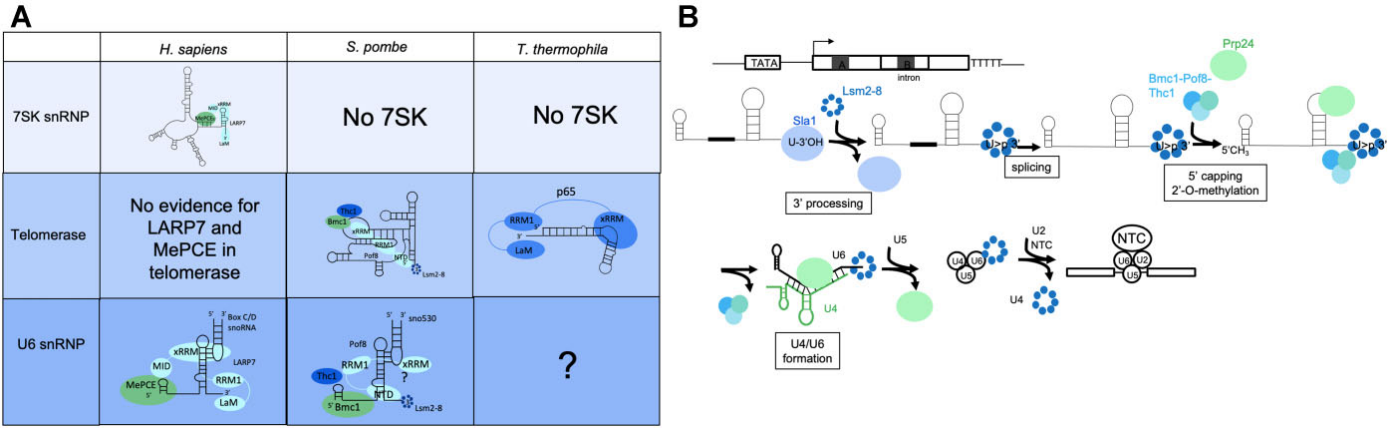
Evolutionary convergence and divergence of Bmc1/MePCE and Pof8/LARP7 in noncoding RNA processing. (**A**) Summary of Bmc1/MePCE and Pof8/LARP7/p65 functions in the 7SK snRNP, telomerase holoenzyme, and U6 snRNP. LaM = La motif, RRM1 = RNA Recognition Motif 1, xRRM = extended RNA Recognition Motif, NTD = N-terminal domain (Lsm2-8-interacting region), MID = MePCE-Interacting Domain. The existence and composition of a U6 snRNP in *T. thermophila* is currently unknown. (**B**) Schematic of the U6 biogenesis pathway in fission yeast. NTC = NineTeen Complex.

Several fungal species, including *S. cerevisiae*, lack both a LARP7 and MePCE homolog (19,20). As deletion or depletion of LARP7/Pof8 or MePCE/Bmc1 does not influence U6 stability in species where this has been investigated (8,20,26,27,34), the function of LARP7 and MePCE family members in U6 biogenesis and function has remained unclear. This work expands our understanding of the evolutionary conservation of LARP7 family members, with shared or unique functions relating to the telomerase, U6, and 7SK RNAs, depending on the species under investigation (Figure 6A). Further links can be drawn between the functional consequences of LARP7- and Pof8-mediated promotion of U6 2′-O-methylation. LARP7 or Pof8 deletion and the subsequent decrease in 2′-O-methylation of U6 results in no functional consequences under standard physiological conditions, but becomes important for maintaining splicing fidelity under heat stress, in the case of human LARP7, and male germ cells in mice (26,27). As the loss of A64 modification alone results in no changes to U6 snRNP assembly, compared to the changes observed upon Bmc1, Pof8, and Thc1 deletion, we anticipate that it is either a combination of the loss of several 2′-O-methylations or the loss of the Bmc1-Pof8-Thc1 U6 snRNP that leads to the slight increase in intron retention at elevated temperatures upon Bmc1 deletion (Figure 5). Future studies aimed at teasing apart this mechanism in mammalian and yeast cells will provide additional insight into the intertwining role of RNA modifications and RNP biogenesis complexes in spliceosome assembly.

We note that the observed heat shock-dependent splicing defect in Bmc1 knockout cells is relatively minor. Previous studies on mammalian LARP7 proposed that LARP7-guided 2′-O-methylation of U6 is not an important factor for splicing as a whole, but rather contributes to splicing robustness (26,27). Although recent reports indicate that alternative splicing in fission yeast may be more widespread than previously thought (63,72,73), splicing complexity in fission yeast is still less than that observed in mammalian cells, which may explain why we do not observe any drastic splicing changes upon Bmc1 deletion. It remains to be determined whether Bmc1 affects other aspects of splicing that have not been tested here, such as splicing efficiency and fidelity, or whether Bmc1-associated defects in splicing might be greater under different stresses. In addition, Bmc1 promotes 2′-O-methylation in the internal stem loop of U6, which does not base pair with the 5′ or 3′ splice site. Thus, modulation of ISL modifications might not be expected to manifest as a robust splicing defect. This is in contrast to what has been reported for the loss of m^6^A in *S. pombe* U6, where affected introns are enriched for an adenosine at the fourth position of the intron, which directly base pairs with the m^6^A (74). While it is surprising that Bmc1, Pof8, and Thc1 deletion have no effect on A41 methylation levels, despite the recovery of snoZ30 in our Bmc1 RIP-Seq, A41 is the only 2′-O-methylation occurring outside the U6 ISL. This suggests that the Bmc1-containing U6 snRNP may only have an important role in guiding ISL modifications, much like LARP7 facilitates the analogous ISL modifications in mammals (26,27).

### Emerging importance of the xRRM in RNA folding and function

Fission yeast, possessing a LARP7 homolog that functions in telomerase like its ciliate counterpart (20,29–34), and U6 2′-O-methylation in an analogous manner to its mammalian homologs, may represent an evolutionary intermediate bridging RNA binding proteins between ciliates and mammals. The 7SK snRNA, which has only been found in animals (75) likely arose independently from the more widely distributed LARP7 and MePCE, suggesting the need for continued studies into 7SK-independent functions for LARP7 and MePCE. Of note, the conservation of the xRRM between fungal, mammalian, and ciliate LARP7 proteins, rather than the La motif (19,20,29–31) may provide a reason explaining the diverse RNA substrates bound by LARP7 homologs, compared to the more well-conserved classes of RNA binding partners of other LARPs across species (19). xRRM-mediated binding to structured stem loops like the telomerase RNA pseudoknot (32), SL4 of 7SK (76), and U6-modifying snoRNAs (27) may be a better determinant than 3′ terminal uridylate stretches for predicting LARP7 binding. The importance of the xRRM in the biogenesis and stability of telomerase RNA, 7SK, and U6 may be linked to its RNA chaperone activity, which has been proposed to have a role in promoting RNA folding (61,62). Our finding that mutation of the xRRM of Pof8 impairs 2′-O-methylation of U6 without disrupting U6 binding (Figure 5D) may provide further evidence that the xRRM has functions beyond U6 binding and raises additional questions as to the mechanism by which RNA chaperones can coordinate snoRNA and target RNA binding to carry out efficient 2′-O-methylation. Importantly, xRRM chaperone activity is not limited to LARP7 family proteins, as the RRM2/xRRM of the human La protein has also been shown to promote RNA folding (77–79).

### New insights into U6 biogenesis in fission yeast

This work also sheds light on the timing of U6 biogenesis steps in fission yeast (Figure 6B). We have previously shown that Lsm2-8 interacts with both mature and intron-containing U6, suggesting that intron removal occurs after 3′ end processing and the switch from La to Lsm2-8 (20,23). Conversely, Bmc1 and Pof8 interact solely with the spliced form of U6 (20). This, coupled with our finding that the Lsm2-8-interacting region of Pof8 is required for the Bmc1-U6 interaction (Figure 5D, E), indicates that Lsm2-8 binding occurs prior to splicing and recruitment of the Bmc1-Pof8-Thc1 complex. Our data indicating that Bmc1 co-purifies with U6-modifying snoRNAs (Figure 1 and Supplementary Figure S2) suggests that U6 then undergoes 5′ capping by Bmc1 and 2′-O-methylation, prior to Bmc1-Pof8-Thc1 dissociation from U6 and U4/U6 di-snRNP assembly mediated by Prp24. Since deletion of Bmc1 or Pof8 results in decreased association of Prp24 with U4 and U6 (Figure 2), the Bmc1-Pof8-Thc1 complex may play a role in the handoff to Prp24. This role may be mediated by xRRM-linked chaperone activity that remodels U6 to better position it to interact with Prp24 and U4. Our finding of a new U6 biogenesis complex thus adds another layer of regulation to spliceosome assembly. Still, it remains unknown whether Bmc1, Pof8, and Thc1 only interact with U6 during its biogenesis, or re-associate with U6 when it is reassembled into the U4/U6 di-snRNP for subsequent rounds of splicing catalysis, although the significant decrease in abundance of the U4-lacking U6 snRNP (Figure 2A) as well as the impaired binding of U4 and U6 to Prp24 upon deletion of any of Bmc1, Pof8 or Thc1 (Figure 2G) may be more consistent with re-engagement of the Bmc1/Pof8/Thc1 complex after dissociation of U6 from the newly spliced pre-mRNA. Indeed, U6 is the only spliceosomal RNA that is released from the post-catalytic spliceosome as a free RNA, rather than a snRNP, and therefore must re-associate with Lsm2-8 and Prp24 to form the U4/U6 di-snRNP with each round of splicing (80). Additionally, our finding of a mono-U6 snRNP containing Bmc1 and Pof8 that promotes internal modifications of U6 is consistent with earlier reports of the human m^6^A methyltransferase METTL16 present in a mono-U6 snRNP with MePCE and LARP7 (81). Since mammalian U6 also undergoes 5′ methyl phosphate capping by MePCE and LARP7-mediated 2′-O-methylation, it will be interesting to examine the interplay between MePCE, LARP7, and METTL16, and how these factors may function in promoting the formation of the U4/U6 di-snRNP in higher systems.

Taken together, this work adds to the growing body of literature on the catalytic-independent functions of RNA modification enzymes (reviewed in (82)). While this raises questions as to the precise function of Bmc1 catalytic activity on the 5′ end of U6, *in vitro* binding assays showed that catalytic activity of the human MePCE promotes 7SK retention following catalysis (83). It remains to be found if this extends to other MePCE/Bmc1 targets like U6, and how U6 snRNP assembly may be regulated in species lacking MePCE/Bmc1 and LARP7 homologs.

## Supplementary Material

gkad563_Supplemental_Files

## Data Availability

The data supporting the findings of this study are available from the corresponding author upon reasonable request. RNA Seq data have been deposited in NCBI’s Sequence Read Archive (SRA) database under BioProject number PRJNA918556.
