# Supplementary material for: The fission yeast methyl phosphate capping enzyme Bmc1 guides 2′-O-methylation of the U6 snRNA
Source: Nucleic Acids Res. 2023 Jul 5;51(16):8805–19. doi: 10.1093/nar/gkad563 (PMC10484740; doi:10.1093/nar/gkad563)
Supplement: gkad563_Supplemental_Files [file gkad563_supplemental_files.zip › Porat et al., 2023 supplementary information.pdf]

Supplementary information for

The fission yeast methyl phosphate capping enzyme Bmc1 guides 2'-O-methylation of the U6 snRNA

Jennifer Porat[1], Viktor A. Slat[2], Stephen D. Rader[2,3], Mark A. Bayfield[1]*

1. Department of Biology, York University, Toronto, Canada
2. Department of Biochemistry and Molecular Biology, University of British Columbia, Vancouver, Canada
3. Department of Chemistry and Biochemistry, University of Northern British Columbia, Prince George, Canada

*Mark A. Bayfield

Email: bayfield@yorku.ca

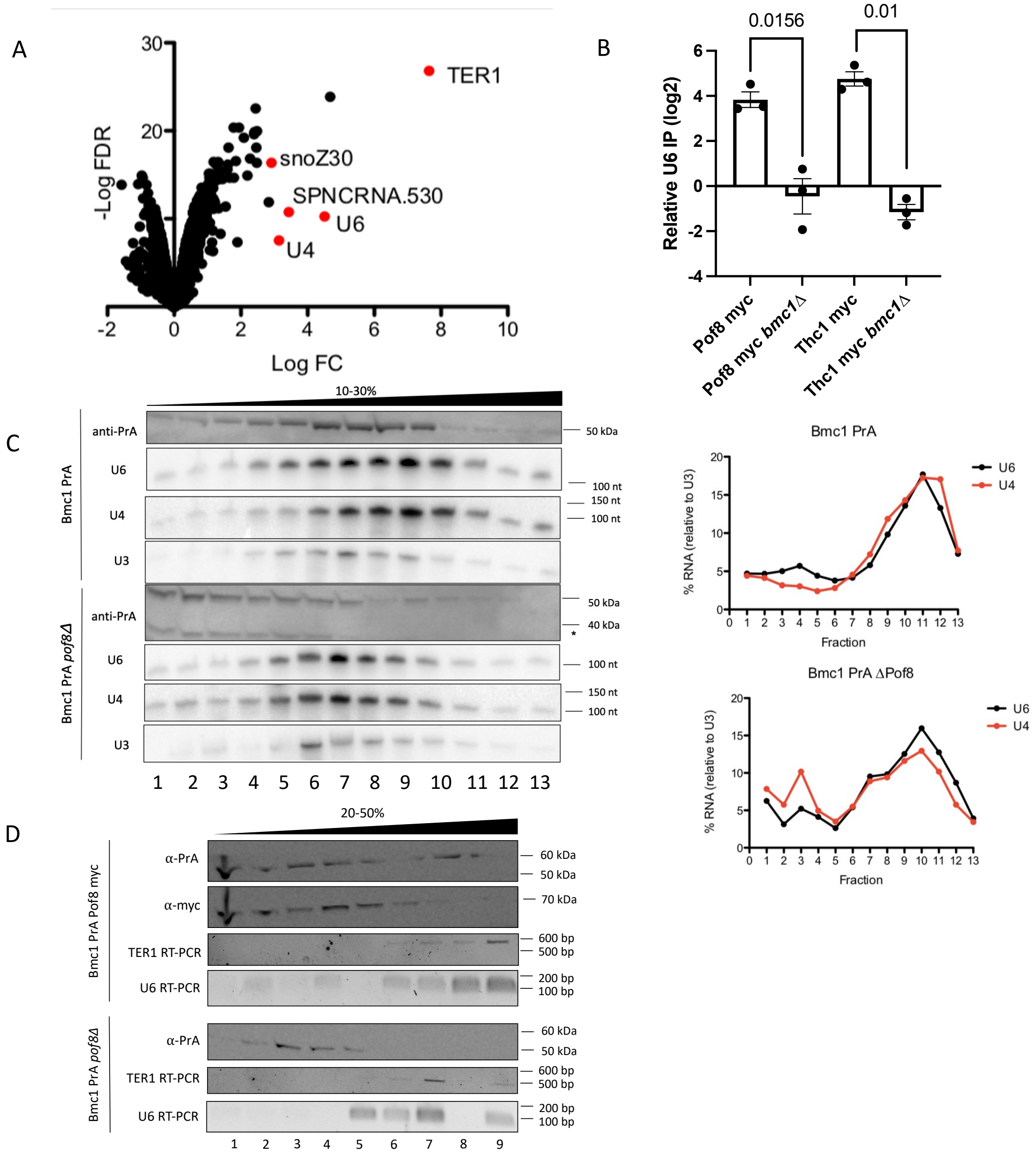

Figure S1: Bmc1, Pof8, and Thc1 cooperate to bind U6 and U6-associated noncoding RNAs

A) Enrichment of Bmc1 PrA-associated transcripts compared to an untagged control (n= 3 biological replicates). Axes represent log2 of fold change (FC) and negative log of false discovery rate (FD) (Benjamini-Hochberg adjusted *P* value ≤0.05). Data taken from (1).

B) qRT-PCR of U6 in Pof8 myc and Thc1 myc immunoprecipitates, normalized to immunoprecipitation from an untagged strain (mean± standard error, two-tailed paired *t* test*)* (n= 3 biological replicates).

C) Glycerol gradient sedimentation of PrA-tagged Bmc1, U4, U6, and U3 from wild type (Bmc1 PrA) and *pof8Δ* strains. Cleavage products are indicated with an asterisk. U4 and U6 signals were normalized to U3 for calculating relative migration in the gradient.

D) 20-50% glycerol gradient sedimentation of PrA-tagged Bmc1, myc-tagged Pof8, TER1, and U6 in wild type (Bmc1 PrA) and *pof8Δ* strains. TER1 and U6 were amplified with 22 and 17 PCR cycles, respectively.

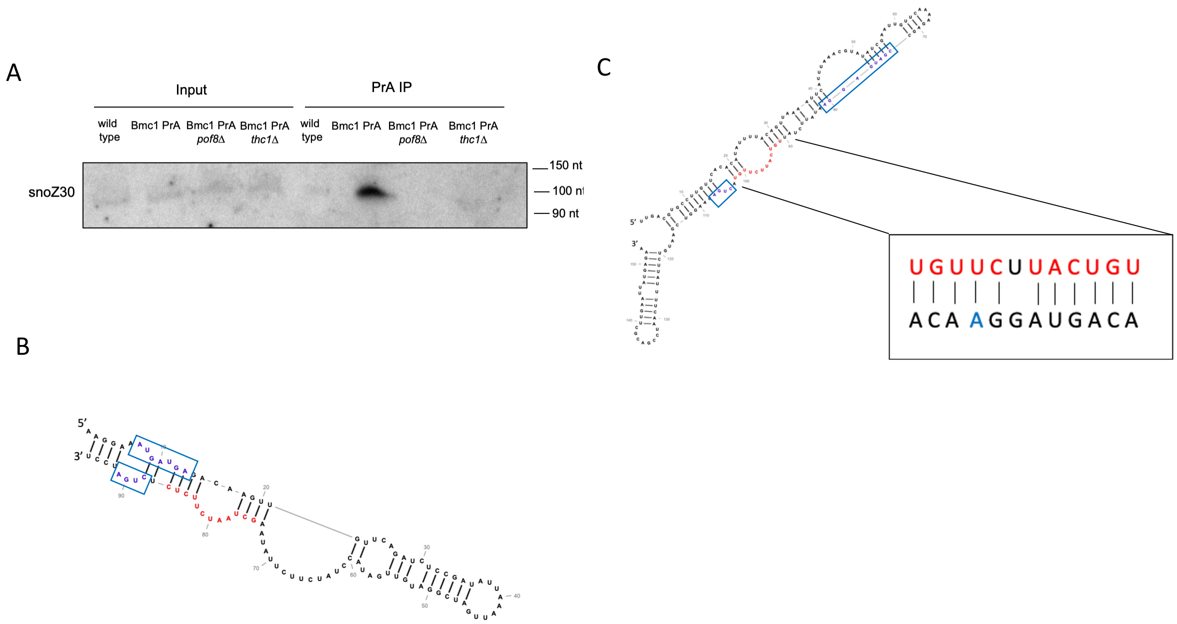

Figure S2: snoZ30 and sno530 are Bmc1-interacting, U6-modifying snoRNAs

A) Northern blot analysis of snoZ30 in total RNA and PrA immunoprecipitates from an untagged strain (wild type) and wild type and knockout PrA-tagged strains.

B) Secondary structure prediction (2) of snoZ30. C and D boxes are indicated in blue and U6-binding site is indicated in red.

C) Secondary structure prediction (2) of sno530. C and D boxes are indicated in blue and U6-binding site is indicated in red. Inset: U6-interacting region, highlighting Watson Crick and non-Watson Crick base pairs with U6 (black). A64 is indicated in blue.

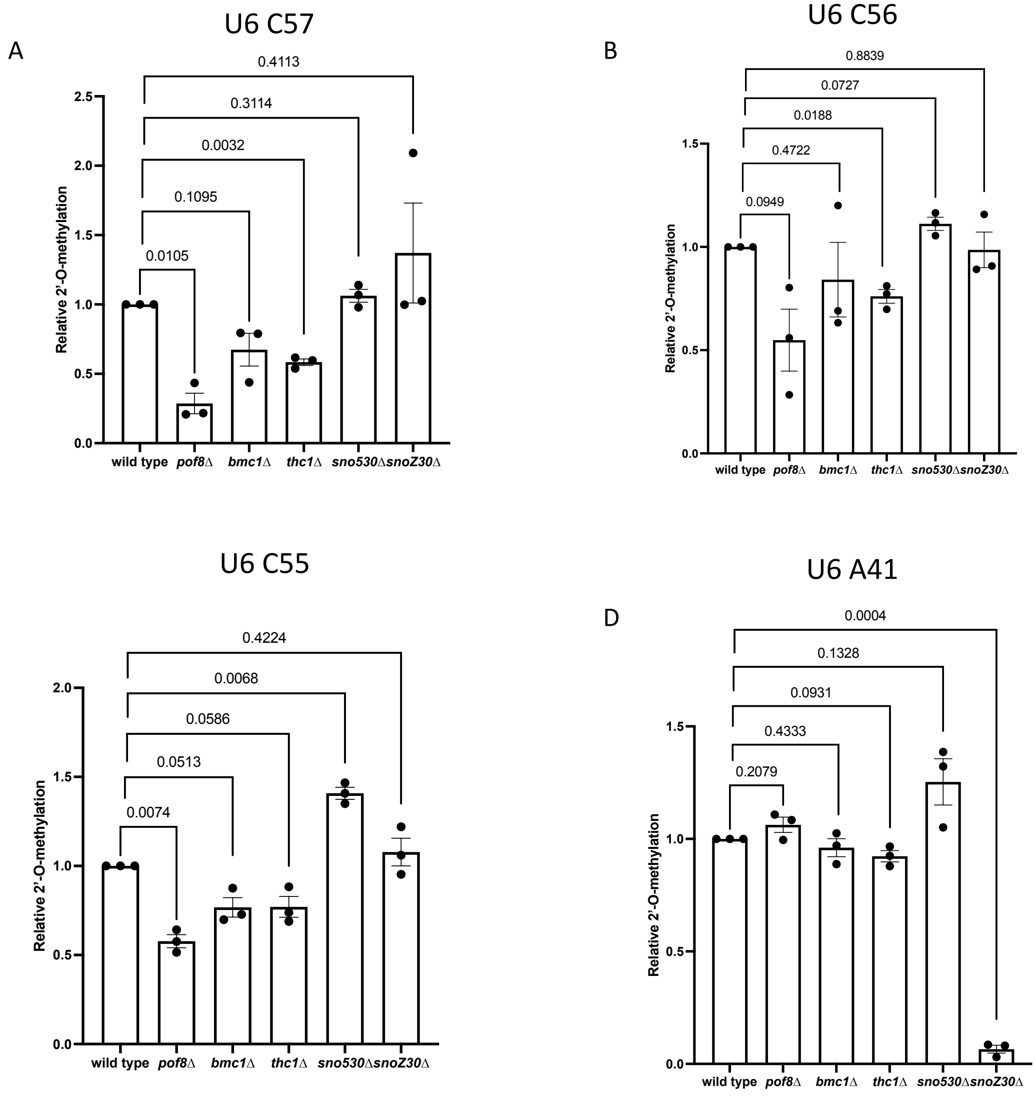

Figure S3: Bmc1, Pof8, and Thc1 influence 2'-O-methylation of U6
Quantification of relative 2'-O-methylation-induced reverse transcriptase stops, compared to a wild type strain, for C57 (A), C56 (B), C55 (C),and A41 (D) (mean± standard error, two-tailed paired *t* test) (n= 3 biological replicates).

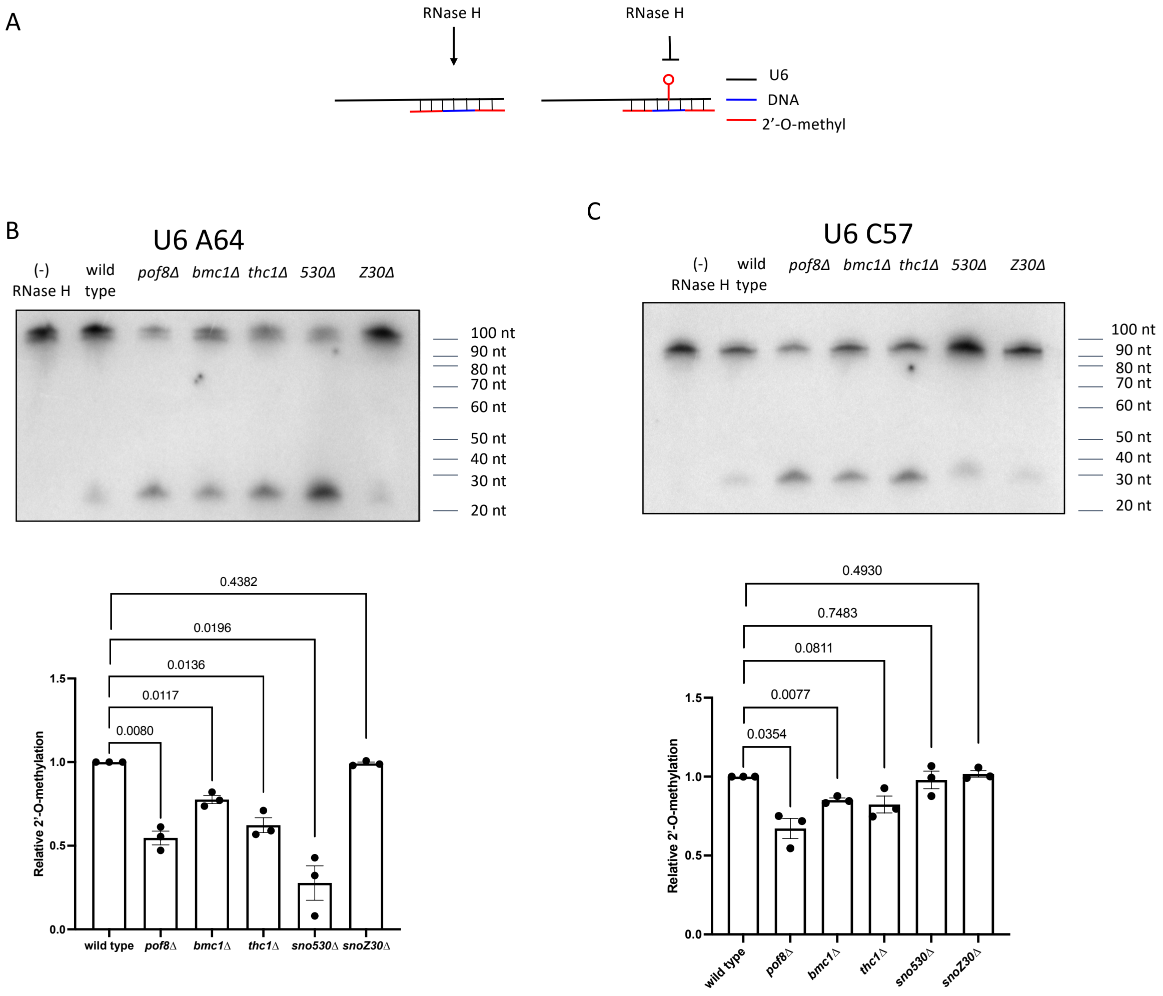

Figure S4: RNase H cleavage validates 2'-O-methylation of U6 at A64 and C57

A) Schematic of RNase H cleavage assay to detect 2'-O-methylations (3, 4).

B-C) Northern blot analysis and quantification of 2'-O-methylation at A64 (B) and C57 (C). Relative modification is expressed as a fraction of the cleaved band relative to total U6 (mean± standard error, two-tailed paired *t* test) (n= 3 biological replicates).

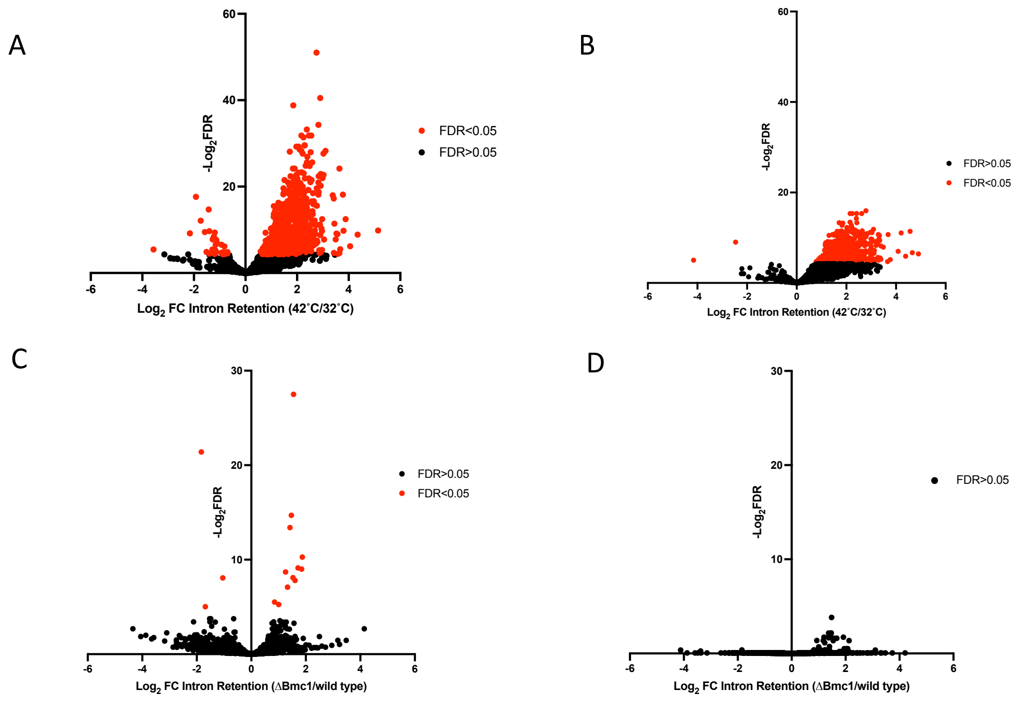

Figure S5: Heat shock and Bmc1 deletion lead to changes in intron retention.
A-B) Changes in intron retention in wild type (A) and *bmc1Δ* (B) strains grown at 32˚C or heat shocked for 15 minutes at 42˚C (n=3 biological replicates). Axes represent log2 of fold change (FC) and negative log2 of false discovery rate (FD) (Benjamini-Hochberg adjusted *P* value ≤0.05). C-D) Changes in intron retention in wild type and *bmc1Δ* strains grown at 32˚C (A) or heat shocked for 15 minutes at 42˚C (B) (n=3 biological replicates). Axes represent log2 of fold change (FC) and negative log2 of false discovery rate (FDR) (Benjamini-Hochberg adjusted *P* value ≤0.05).

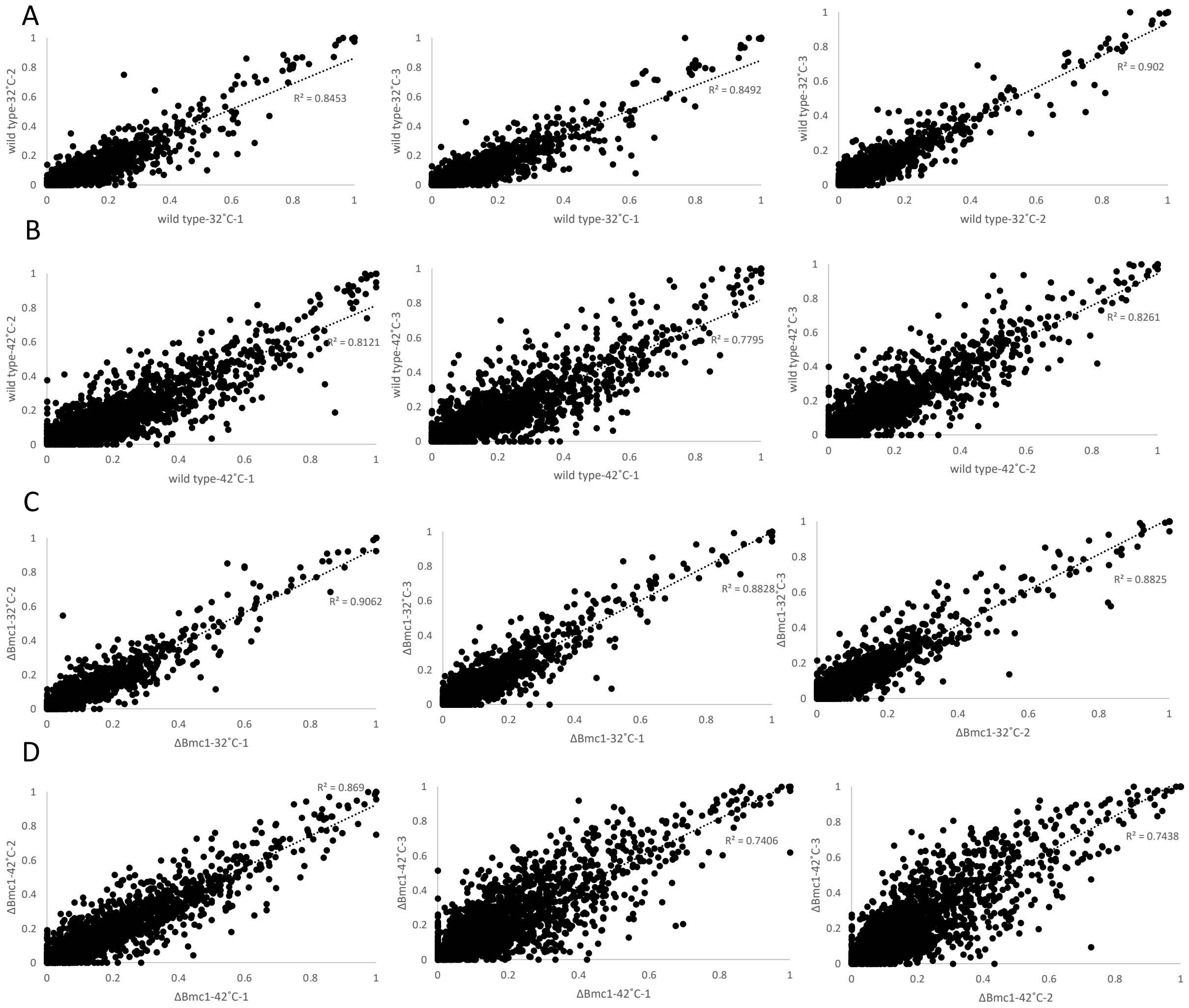

Figure S6: Correlations between replicates for RNA-Seq of wild-type and *bmc1Δ* strains with and without heat shock.

Intron retention values for wild type RNA Seq samples grown at 32˚C (A) and 42˚C (B) and ΔBmc1 cells grown at 32˚C (C) and 42˚C (D). R^2 values are displayed.

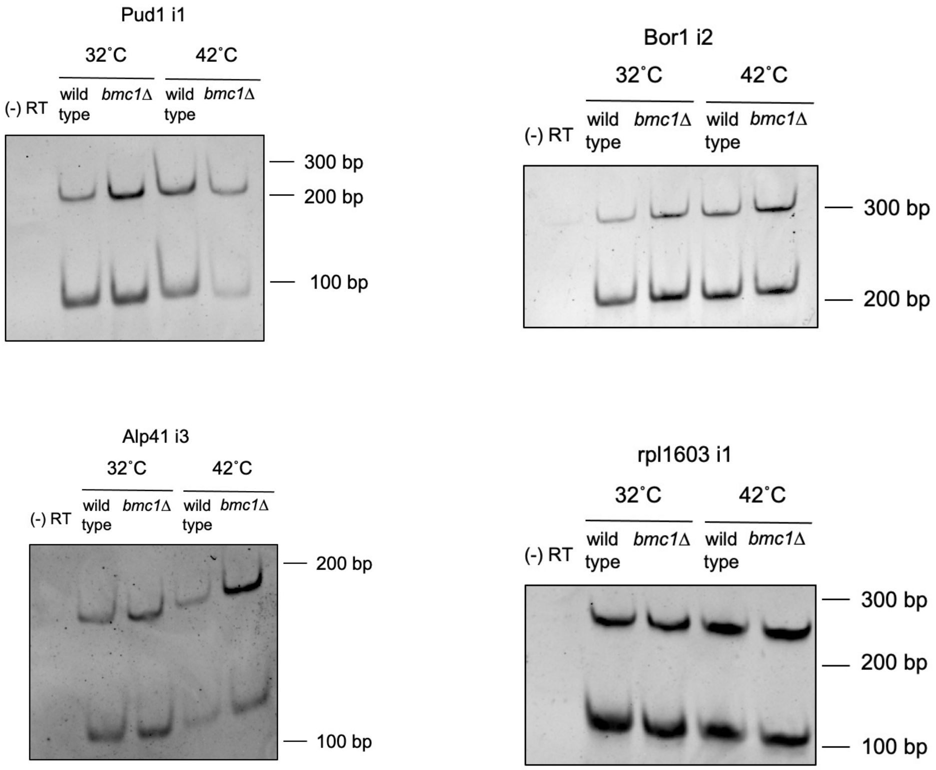

Figure S7: Semi-quantitative RT-PCR validation of heat shock- and Bmc1-sensitive intron retention events.

Representative gels (from n=3 biological replicates) contributing to quantifications in figure 5B.

Table S1: Primer sequences for the creation of tagged and knockout *S. pombe* strains

Primer	Sequence
5’ Thc1 myc SalI For	5’ GCGCGTCGACATATTCATTGAAGTTGTAGTTTTAGCTTATTTGGTACCA AAAG 3’
5’ Thc1 myc BamHI Rev	5’ GCGCGGATCCGACCTGAAGTCAAGAAATTTTGTAGTGGAATAATTCTT TTC 3’
3’ Thc1 myc SacI For	5’GCGCGAGCTCACAGATAAATTAGAACACAGCTTAAACTTACCGGAAAAA TTAATC 3’
3’ Thc1 myc SacII Rev	5’ GCGCCCGCGGTTAGATAGCCATGAATAAATAATATCAAGAATATAATC ATCTAAGC 3’
5’ Prp24 myc XhoI For	5’ CGCGCTCGAGCCTTAGCTAAAAGCTTTGAGACTACTGAGTCAAATAAA ATG 3’
5’ Prp24 myc BamHI Rev	5’ GCGCGGATCCGTTTTAAAAACATTTTCCTAAAATCATCGTTGCTTTTAG GTGCATC 3’
3’ Prp24 myc SacI Rev	5’ GCGCGAGCTCTCAGCAATAATTAGATTGAATGATTAAAAAAATATTGAA AACC 3’
3’ Prp24 myc SacII Rev	5’ CGCGCCGCGGTGAATATCATTAAAACTCCTTCATTTACTCTTGATGAAA AAATTCAAGAGTC 3’
5’ Thc1 KO SalI For	5’ GCGCGTCGACATAATAACTTTGCTTACGATTAATAGACAAATGAATGC TG 3’
5’ Thc1 KO BglII Rev	5’ GCGCAGATCTTCTTCAAAACTTTTGGTACCAAATAAGCTAAAACTACA A 3’
3’ Thc1 KO ClaI For	5’ GCGCATCGATACAGATAAATTAGAACACAGCTTAAACTTACCGGAAAA A 3’
3’ Thc1 KO SacII Rev	5’ GCGCCCGCGGCTTTACTTAAACAGAGAAAAAAAAAACATCTGGAGA C 3’
5’ sno530 KO SalI For	5’ GCGCGTCGACGCATGTAAACTGTTTCACGACTTAACGGATCATATG G 3’
5’ sno530 KO BglII Rev	5’ GCGCAGATCTATTGACAACAATCAACTGCGTGTTTTATTTACTTTTAAG A 3’
3’ sno530 KO ClaI For	5’ GCGCATCGATTCATACAAAAAACTGAGGCTATTTGCTTTAACTGTAGC T 3’
3’ sno530 KO SacII Rev	5’ GCGCCCGCGGTGAACCTCAAGTCCCGCAAAATACTTCCAATAATAATT TG 3’
5’ snoZ30 KO BamHI For	5’ GCGCGGATCCATTTCTTTGCATCCTTTAATATACTTTCCAATTCATTAA G 3’
5’ snoZ30 KO AscI Rev	5’ GGCGCGCGCGCCCTCCTGGTCGACTTTATGGAACTAAAGGGTTACAA TTG 3’
3’ snoZ30 KO SacI For	5’ GCGCGAGCTCATCAAAGTAACTTTCTCGGAGAAAGGTCGAGCAATTG TAAT 3’
3’ snoZ30 KO ClaI Rev	5’ GCGCATCGATACAAATAAATACGATTAGTCTTAAGTTAATTTAGCACAA AGATTTAAAGTC 3’

Table S2: List of yeast strains used in this study

Figure	Strain	Description	Full genotype	Source
1a, 1c, 2, 3, S1a, S1b, S2a, S3, S4, S5	y12088	wild type	*h+ ura4-D18 leu1-32*	Lab stock
1, 2c, S1a, S2a	yJP001	*bmc1-PrA*	*h- ura4-D18 bmc1^{+}::bmc1-PrA- kanMX6*	Porat et al., 2022
1a, 1b, S2a	yJP011	*bmc1-PrA pof8Δ*	*h- leu1-32 ura4-D18 his3-D1 bmc1^{+}::bmc1-PrA-kanMX6 pof8Δ::natMX6*	Porat et al., 2022
1a, S2a	yJP027	*bmc1-PrA thc1Δ*	*his3-D1 bmc1^{+}::bmc1-PrA-kanMX6 thc1Δ::bleMX6*	This study
1c, 2a, 2d, 5, S3, S4	TN12118a	*pof8Δ*	*h- leu1-32 ura4-D18 his3-D1 pof8Δ::natMX6*	Mennie et al., 2018 and Porat et al., 2022
1c, 2a, 2d, 3, 4, S3, S4	yJP022	*bmc1Δ*	*h+ ura4-D18 leu1-32 bmc1Δ::bleMX6*	Porat et al., 2022
1c, 2a, 2d, S3, S4	yJP026	*thc1Δ*	*h+ ura4-D18 thc1Δ:: bleMX6*	This study
1c, 2a, 2d, S3, S4	yJP030	*sno530Δ*	*h+ ura4-D18 sno530Δ::bleMX6*	This study
1c, S3, S4	yJP031	*snoZ30Δ*	*h- ura4-D18 snoZ30Δ::bleMX6*	This study
S2b, S2c	AM16932	*pof8-myc*	*h- leu1-32 ura4-D18 his3-D1 pof8^{+}::13myc-kanMX6*	Mennie et al., 2018
S2b, S2c	yJP023	*pof8-myc bmc1Δ*	*leu1-32 ura4-D18 his3-D1 pof8^{+}::13myc-kanMX6 bmc1Δ::bleMX6*	Porat et al., 2022
S2b, S2c	yJP029	*thc1-myc*	*h- leu1-32 ura4-D18 his3-D1 thc1^{+}::13myc-kanMX6*	This study
S2b, S2c	yJP046	*thc1-myc bmc1Δ*	*leu1-32 ura4-D18 his3-D1 thc1^{+}::13myc-kanMX6 bmc1Δ::bleMX6*	This study
2g	yJP048	*prp24-myc*	*h- leu1-32 ura4-D18 his3-D1 prp24^{+}::13myc-kanMX6*	This study

2g	yJP049	*prp24-myc pof8Δ*	*leu1-32 ura4-D18 his3-D1 prp24$^+$::13myc-kanMX6 pof8Δ::natMX6*	This study
2g	yJP050	*prp24-myc bmc1Δ*	*leu1-32 ura4-D18 his3-D1 prp24$^+$::13myc-kanMX6 bmc1Δ::bleMX6*	This study

Table S3: List of primer and RNA sequences used in this study

Figure	Probe	Sequence
1a, S1b, S1d	U6 RT-PCR For	5’ CGGATCACTTTGGTCAAATTG 3’
	U6 RT-PCR Rev	5’ CTCTCAATGTCGCAGTGTCATC 3’
1a, 4f	530 RT-PCR For	5’ ATGAGGAATATTCTATTGTCATTC 3’
	530 RT-PCR Rev	5’ AACAATTCGATATACGTTTAAATG 3’
1b, 1c, 2a, 2c, 2d, 2g, 4b, 4e, 5b, 5d, S2c, S2d	U6 northern, primer extension, solution hybridization	5’ AATGGGTTTTCTCTCAATGTCGCAG 3’
1b, 2a, 2d, 2g	U4 northern and solution hybridization	5’ GTTGGAGCGGTCAGGGTAATAGT 3’
S2a	snoZ30 northern	5’ GGAGATCTGAACAACTTGTCTCATC 3’
2a	U1 northern	5’ GCTGCAGAAACTCATGCCAGGTAAGT 3’
2a	U2 northern	5’ TGCCAGTAGTGCAATAGCAAGAACAC 3’
2a	U3 northern	5’ ACACGTCAGAAAACACCAGCTGCCC 3’
2a	U5 northern	5’ GATTACAAAAACTATACAGTCAAATTAGCAC 3’
2f	Unmodified U6 oligo	5’ rUrGrGrCrCrCrCrUrGrCrArCrArArGrGrArUrGrArCrA 3’
2f	A64m U6 oligo	5’ rUrGrGrCrCrCrCrUrGrCrArCrAmArGrGrArUrGrArCrA 3’
2f	U4 oligo	5’ rArUrCrUrUrUrGrUrGrCrArCrGrGrGrUrArU 3’
S4b	U6 A64 RNase H chimeric oligo	5’ mUmCCTTGmUmGmCmAmGmGmGmGmCmCmAmU 3’
S4c	U6 C57 RNase H chimeric oligo	5’ mGmCAGGGmGmCmCmAmUmGmCmUmAmAmUmC 3’

Dataset S1 (separate file). Intron retention (IR) ratio values and intron features for wild type (y12088) and ΔBmc1 RNA Seq analysis at 32˚C and 42 ˚C. Introns with more than 4 reads supporting splicing in all biological replicates are included.

Supplemental References

1. Porat,J., El Baidouri,M., Grigull,J., Deragon,J.-M. and Bayfield,M.A. (2022) The methyl phosphate capping enzyme Bmc1/Bin3 is a stable component of the fission yeast telomerase holoenzyme. *Nat. Commun.*, **13**, 1277.
2. Gruber,A.R., Lorenz,R., Bernhart,S.H., Neuböck,R. and Hofacker,I.L. (2008) The Vienna RNA Websuite. *Nucleic Acids Res.*, **36**, W70–W74.
3. Yu,Y.T., Shu,M. Di and Steitz,J.A. (1997) A new method for detecting sites of 2′-O-methylation in RNA molecules. *RNA*, **3**, 324–331.
4. Calo,E., Flynn,R.A., Martin,L., Spitale,R.C., Chang,H.Y. and Wysocka,J. (2015) RNA helicase DDX21 coordinates transcription and ribosomal RNA processing. *Nature*, **518**, 249–253.
